# The Emergence and Fate of Horizontally Acquired Genes in *Escherichia coli*

**DOI:** 10.1371/journal.pcbi.1000059

**Published:** 2008-04-11

**Authors:** Mark W. J. van Passel, Pradeep Reddy Marri, Howard Ochman

**Affiliations:** 1 Department of Biochemistry and Molecular Biophysics, University of Arizona, Tucson, Arizona, United States of America; 2 BIO5 Institute, University of Arizona, Tucson, Arizona, United States of America; ETH Zürich, Switzerland

## Abstract

Bacterial species, and even strains within species, can vary greatly in their gene contents and metabolic capabilities. We examine the evolution of this diversity by assessing the distribution and ancestry of each gene in 13 sequenced isolates of *Escherichia coli* and *Shigella.* We focus on the emergence and demise of two specific classes of genes, ORFans (genes with no homologs in present databases) and HOPs (genes with distant homologs), since these genes, in contrast to most conserved ancestral sequences, are known to be a major source of the novel features in each strain. We find that the rates of gain and loss of these genes vary greatly among strains as well as through time, and that ORFans and HOPs show very different behavior with respect to their emergence and demise. Although HOPs, which mostly represent gene acquisitions from other bacteria, originate more frequently, ORFans are much more likely to persist. This difference suggests that many adaptive traits are conferred by completely novel genes that do not originate in other bacterial genomes. With respect to the demise of these acquired genes, we find that strains of *Shigella* lose genes, both by disruption events and by complete removal, at accelerated rates.

## Author Summary

Changes in genetic repertoires can alter the adaptive strategy of an organism, especially in bacteria, in which genes are continually gained and lost. Mapping the gains and losses of genes in the densely sequenced clade of *Escherichia coli* and *Shigella* shows that these genomes harbour two types of acquired genes: HOPs, which are those acquired genes with homologs in distantly related bacteria; and ORFans, which are genes without any known homologs. Surprisingly, the two classes of acquired genes display very different patterns of gain and loss. HOPs are acquired more frequently, though they rarely persist in the recipient genomes. In contrast, ORFans are much more likely to be maintained over evolutionary timescales, suggesting that despite their unknown origins, they will more often confer novel and beneficial traits to the recipient genome.

## Introduction

The wide variation in bacterial genome sizes was originally detected in the 1960s by DNA reassociation analyses [1]. And because bacteria have gene dense chromosomes, the differences in genome sizes implied that there were likely to be vast differences in the gene contents of bacterial species. With the current availability of hundreds of complete genome sequences, it is now possible to establish exactly which genes are present in, as well as those that are absent from, a genome. Among sequenced bacterial genomes, gene sets vary over 40-fold, ranging from 182 genes in the gammaproteobacterial symbiont *Carsonella ruddii* [2] to almost 8000 genes in the soil-dwelling acidobacterium *Solibacter usitatus* (jgi.doe.gov).

The wide variation in genome sizes and gene contents can also be observed between strains within individual bacterial genera or species. For example, isolates of *Frankia* that are more than 97% identical in their rRNA sequences–the conventional cutoff value for a bacterial species–can differ by as many as 3500 genes, which represents nearly half of their 7.5 Mb genome [3]. Even among bacterial strains of similar genome sizes, there can be substantial differences in gene repertoires [4]. Unlike mammals, in which only about 1% of the genes in a genome are unique to a taxonomic order (*e.g*., mouse vs. human [5]), the gene contents of bacterial genomes can change rapidly over relatively short evolutionary distances.

The generation of novel gene repertoires is a consequence of the ongoing processes of gene acquisition and gene loss [6]–[10]. Although several mechanisms can generate new genes [6],[11],[12], the novel gene sets observed in closely related bacterial strains result largely from gene transfer from distant sources, as duplications and gene rearrangement only rarely produce entirely unique genes in the short timescales in which bacterial gene sets evolve. Although homolog searches indicate that many genes arise from lateral transfer from other bacteria, most bacteria also contain genome-specific sets of genes that lack any homologs in the known databases (termed “ORFans”) [4],[13],[14]. Counteracting the augmentation of bacterial genomes by gene acquisition, gene loss occurs both through large-scale deletions [15] as well as by smaller changes that erode and inactivate individual genes [7],[9],[16]. As observed for acquired sequences, prokaryotes also contain genome-specific sets of inactivated genes (*i.e.*, pseudogenes), which can comprise up to 41% of their annotated genes [17].

Taken together, these lineage-specific gene repertoires indicate the need to monitor bacterial genome dynamics–*i.e.*, the manner in which genes are gained and lost–over short evolutionary timescales. To this end, comparisons of closely related strains of *Bacillus* [18], *Staphylococcus aureus* [19] and *E. coli* [7],[20] have shown that gene acquisitions are prevalent at the tips of the phylogeny and that recently acquired genes seem to evolve more quickly. However, few studies have examined the fate of these genes within a bacterial lineage or have asked how many or which classes of genes, once acquired, are maintained, disrupted or removed from a genome. We address these questions by assessing the differences in gene repertoires among 13 sequenced strains of *E. coli*/*Shigella* clade. These strains are closely related, yet display substantial differences in genome size and gene content [21],[22], allowing us to pinpoint the introduction and persistence of genes in the lineages leading to these genomes.

## Results

### The *E. coli* Species Tree

**Figure 1:**
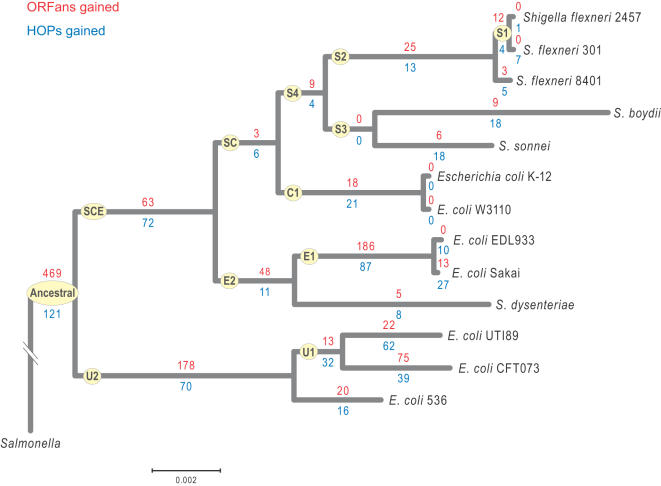
Phylogeny of *E. coli* and *Shigella* strains showing the numbers of ORFan genes (red) and HOP genes (blue) gained on each branch. Yellow-shaded ovals contain the letter abbreviations used to refer to ancestral lineages and clades. The clades S4 and U1 have bootstrap values of 95%, while the rest have a bootstrap value of 100%.

We reconstructed the phylogeny of 13 sequenced strains of *E. coli* and *Shigella* species based on the concatenated sequences of 169 conserved, single-copy genes. The relationships and branching orders are well-resolved, well-supported, and congruent with previous studies [23]. The overall branching order of the resulting phylogeny is very similar to those based on other characters or for more limited sets of sequenced strains [24]–[26]: the uropathogenic *E. coli* (UPEC) form a monophyletic cluster at the base of the tree, and *Shigella* strains are polyphyletic, with a major lineage derived from the clade containing *E. coli* K-12 [27]. Based on this tree, we delineated 12 monophyletic clades of varied phylogenetic depths (of which we designate the corresponding ancestral branches as S1 to S4, SC, SCE, C1, E1, E2, U1, U2 and the ancestral branch, Figure 1), which were used to trace the evolutionary history of all genes in these 13 genomes.

### Gene and Gene Family Distributions

By identifying the homologs of genes from the 13 *E. coli* and *Shigella* strains in each of 367 microbial genomes, and by mapping the gene distributions in a phylogenetic context, we could infer the ancestry (vertical or horizontally acquired) and dynamics (incidence of acquisition or loss) of genes among strains. Acquired genes were classified into two categories: ORFans, which are genes that have no homologs outside of the analyzed *E. coli* and *Shigella* strains, and HOPs, which are genes that have homologs outside of the analyzed *E. coli* and *Shigella* genomes but are not ancestral to all taxa containing the gene or whose phylogenetic distributions can not be most parsimoniously reconstructed solely through gene loss events.

From the 13 sequenced strains within the *E. coli/Shigella* clade, we identified a total of 1443 ORFan gene families and 652 HOP gene families (a family is a group of homologs). Gene family sizes ranged from one gene, for ORFans or HOPs present in a single genome (representing 11% and 32% of the total number of families, respectively) to 13 genes, for ORFans or HOPs with homologs present in all 13 genomes (representing 13% and <1% of the total number of families, respectively). We inferred the branch on which ORFans and HOPs originated by reconstructing the most parsimonious series of events that would give rise to their present-day distributions. By this approach, all HOPs could be assigned to a particular clade, but only 1177 of the 1443 ORFan families were assigned unequivocally, and together these constitute the set considered in subsequent analyses.

Only 8 ORFans (<1%) could be classified to a particular COG category, whereas 151 (23%) of the HOP families could be assigned to a COG other than ‘poorly characterized’: these included Metabolism (10%), Cellular Processes and Signaling (8%, mostly in the category Cell Wall/Membrane/Envelope Biogenesis) and Information Storage and Processing (5%) (Supplementary Table S1).

The numbers of acquired ORFans and HOPs vary substantially across strains and lineages (Figure 1), with the largest difference occurring in the gene set acquired by the ancestor to all tested strains in which ORFans are approximately four times more common than HOPs. This is in contrast to genes confined to a single *E. coli* or *Shigella* genome, where we identify ∼40% more HOPs than ORFans. This difference is not affected by the fact that 20% of ORFans could not be placed onto a specific branch, because singleton ORFans are among the easiest genes to assign. Taken together, these distributions suggest that HOP genes originate more frequently, but ORFans are more likely to persist.

### Characteristics of the Acquired Genes

Overall, ORFans constitute between 9% and 14% of the protein coding genes per genome, and HOPs account for at most 5% of the protein coding genes per genome. Cumulatively, ORFans outnumber HOPs; however, HOPs represent a larger proportion of the acquired DNA in all strains as they are, on average, longer than ORFans (853 bp vs. 308 bp respectively) (Table 1). There is an association between genome size and the amount of ORFan and HOP-derived DNA (r^2^ = 0.75 and r^2^ = 0.72, respectively) per genome; however, it is not simply a matter that the strains with the largest genomes have acquired the most DNA. For example, *Shigella dysenteriae* and *E. coli* EDL933 have gained identical amounts of DNA from ORFans and HOPs despite an 800 kb difference in their genome sizes.

**Table 1:** Amounts of DNA gained and lost by strains of *E. coli* and *Shigella* since their last common ancestor.

	DNA Gained (kb)		DNA Lost (kb)		
	ORFans	HOPs	ORFans	HOPs	Net Gain
*S. flexneri* 301	99	232	44	126	161
*S. flexneri* 2457	106	237	52	135	156
*S. flexneri* 8401	111	218	59	116	154
*S. boydii*	105	211	57	108	152
*S. sonnei*	90	201	47	95	149
*S. dysenteriae*	129	205	68	112	154
*E. coli* K-12	72	152	27	52	146
*E. coli* W3110	72	152	27	52	146
*E. coli* EDL933	129	205	15	37	282
*E. coli* Sakai	130	225	13	45	297
*E. coli* UTI89	82	141	16	16	191
*E. coli* CFT073	106	135	25	18	199
*E. coli* 536	81	103	22	23	139

ORFans are more A+T-rich than HOPs (44% vs. 47% G+C, respectively), and such differences in base composition are evident along most lineages (Supplementary Figure S1). When examining a single lineage at increasing phylogenetic depths, there is no clear trend towards increased G+C contents, G+C content of the third codon position or increased gene lengths of ORFans or HOPs with duration in the *E. coli* genome, although this has been observed previously for acquired genes assessed over substantially longer evolutionary timescales [20]. This indicates that the elapsed time since the divergence of the 13 tested strains from their common ancestor has been insufficient to adjust acquired genes to the nucleotide composition of their host genome.

Recently acquired ORFans and HOPs occur more often in multigene clusters than do those assigned to older branches. For example, in *E. coli* CFT073, which contains the largest numbers of both ORFans and HOPs, about half of the ORFans confined to this strain are adjacent to another ORFan of the same age. Going back to the next branch that subsumes this strain (U2), only a third of the ORFans reside next to another ORFan; and among those ORFans originating in the ancestor to the *E. coli/Shigella* clade, only 14% are situated next to another ORFan. The average cluster sizes of ORFans along these three branches are 1.59, 1.34, and 1.09 genes, indicating that ORFan genes are gained in clusters that subsequently shrink through fragmentation and gene loss. For the same lineages, a similar trend is observed for HOPs, although it is not as pronounced (with 1.34, 1.34 and 1.21 genes per cluster for singleton, U2-specific and ancestral HOPs, respectively). This decrease in gene cluster size is not due to the preferential insertion of new genes near older acquired genes, as we analyzed the cluster sizes of sets of ORFans and HOPs per introgression event (*i.e.*, those originating on the same internal branch). A few of the clustered ORFans were located near genes of known phage functions, but a recent exhaustive study into viral ORFans has suggested that phages may play a lesser role in transferring ORFans to prokaryotes than previously thought [28].

### Acquisition Rates of ORFans and HOPs

Since the split from their common ancestor, the 13 *E. coli* and *Shigella* species have accumulated between 180 and 350 kb of foreign DNA per strain (Table 1). Aside from these additions, each of these strains has also lost between 30 and 190 kb of DNA that has been acquired and maintained in other strains. The two EHEC strains (*E. coli* EDL and Sakai) show the highest net gain of DNA, whereas the *Shigella* strains, *E. coli* K-12 and W3110 show the lowest.

To compare the rates at which lineages vary in rates of DNA gain and loss, we calculated the amounts of DNA acquired and lost in relation to the branch lengths in the tree relating the 13 tested genomes. The rates on individual branches indicate that closely related strains can differ by over two orders of magnitude in the rates at which newly acquired DNA is gained and retained (*E. coli* Sakai vs. *S. dysenteriae*) but less than 20-fold in the rates at which such DNA is lost (*E. coli* EDL933 vs. *S. boydii*) (Supplementary Table S2). It should be noted that branch lengths can also vary for other reasons (such as variation in substitution rates and differing rates of recombination), but these are most likely compensated due to the extensive gene set employed here.

Gene acquisition rates for both ORFans and HOPs are higher on the internal branches leading to the EHEC and UPEC strains, and in contrast, rates of loss for acquired DNA are highest on all branches descending from the SC ancestor leading to the *Shigella* species. Taken together, strains that gain the lowest amounts of DNA, lose the highest amounts of acquired DNA with the result that their genomes have lower numbers of unique genes.

### Dynamics of Recently Acquired Genes

There has been a continual gain and loss of ORFans and HOPs during the evolution and diversification of *E. coli* (Supplementary Figures S2 and S3), and based on the distribution of ORFans and HOPs in the 13 tested genomes, HOPs have a higher rate of origination, but ORFans are more likely to be retained.

Since many, possibly most, genes are transient and not present in any contemporary genome, it is not possible to monitor the full complement of genes that are gained and lost in these lineages by comparing their present-day gene repertoires. However, the patterns of retention of genes assigned to evolutionary lineages of different ages offer a glimpse into the fate of acquired sequences. Among genes that originated in the ancestor to all 13 strains examined, 78% (61% of the ORFans and 95% of the HOPs) were lost in one or more of the descendant lineages, whereas 96% (95% of the ORFans and 97% of the HOPs) of the genes acquired on the next older branch, SCE, were lost. Overall, genes acquired on the ancestral branch have higher retention rates than those genes acquired on more recent branches.

Combining the numbers of ORFans and HOPs, *Shigella* spp. (including *S. dysenteriae*) show significantly lower retention rates compared to *E. coli* strains (65% vs. 88%; *p*<0.01), which is not surprising since *Shigella* species have the highest rates of loss of recently acquired genes. The lower retention rates in *Shigella* spp. result from both significantly more gene inactivations (11% vs. 5% in *E. coli*, *p*<0.01) and gene losses (24% vs. 8% lost in *E. coli*, *p*<0.01), and though disruptions occur to a similar extent in both sets of acquired genes, HOPs are more frequently lost than retained as inactivated genes (Table 2, Supplementary Figure S3). Also, ORFans are inactivated predominantly by truncations, whereas HOPs are more often disrupted by insertion sequences (Supplementary Table S3).

**Table 2:** Fate of acquired genes in strains of *E. coli* and *Shigella* spp.

	ORFans						HOPs					
Strain	Intact (n)*	ψ Genes (n)	Lost (n)	Intact (%)*	ψ Genes (%)	Lost (%)	Intact (n)	ψgenes (n)	Lost (n)	Intact (%)	ψ Genes (%)	Lost (%)
*S. flexneri* 301	447	60	74	77	10	13	104	26	91	47	12	41
*S. flexneri* 2457	440	70	71	76	12	12	101	32	95	44	14	42
*S. flexneri* 8401	388	91	93	68	16	16	98	22	103	44	10	46
*S. boydii*	390	55	108	71	10	20	97	17	100	45	8	47
*S. sonnei*	407	47	96	74	9	17	121	28	72	55	13	33
*S. dysenteriae*	402	62	121	69	11	21	99	16	97	47	8	46
*E. coli* K-12	468	39	46	85	7	8	161	9	50	73	4	23
*E. coli* W3110	469	37	47	85	7	9	161	9	50	73	4	23
*E. coli* EDL933	717	33	16	94	4	2	255	1	45	85	0	15
*E. coli* Sakai	735	27	17	94	3	2	268	3	46	85	1	15
*E. coli* UTI89	628	28	26	92	4	4	254	2	29	89	1	10
*E. coli* CFT073	658	37	40	90	5	5	230	10	22	88	4	8
*E. coli* 536	585	50	28	88	8	4	178	8	21	86	4	10

*: (n) denotes the total number of genes in a particular class, and (%) is the percentage of the total number of ORFans or HOPs in a genome.

Although pseudogenes have been shown to be largely genome-specific [7],[9],[16], it was expected that some would be retained in multiple lineages over the short evolutionary time-span examined in this study. However, more than half of the inactivated ORFans and HOPs exist only in a single genome, whereas their functional homologs are usually present in several genomes (data not shown). Similarly, over half of the losses of acquired genes are also genome specific (*i.e.*, losses of the only member of a gene family), confirming the high turnover rate observed for inactivated DNA.

## Discussion

Gene gain and loss are ongoing processes in microbial genomes, resulting in a diversity in genome sizes, even among closely related strains within a bacterial species [3],[29]. By comparing the genome contents of sequenced representatives of the *E. coli/Shigella* clade, and by mapping the phylogenetic distribution of every gene present in these genomes, we find that the rates of change in novel genes can differ over 200-fold between strains and lineages. Moreover, genes of different phylogenetic origins arise and persist at very different rates. For example, ORFan genes, *i.e*., those with no homologs outside of the group of bacteria examined, emerge less frequently than do genes originating by acquisition from other bacteria (termed “HOPs”), but are, on average, about eight times more likely to be maintained. Of the genes acquired on the ancestral branch, nearly 39% of the ORFans, but only 5% of HOPs, are present in all 13 genomes indicating that they now provide functions integral to all strains.

The difference in the persistence of ORFans and HOPs is surprising because those genes acquired from other bacteria (*i.e.*, HOPs) typically encode functional proteins in the donor and could be immediately useful to the recipient, whereas the ORFans, whose origins are less certain, have probably never served a function in a cellular genome prior to their acquisition. The disparity in the types of properties conferred by these two classes of genes is supported by their assignment to known functional categories: whereas nearly a quarter of the HOPs could be designated a COG category, less than 1% of ORFans could. Although ORFans are often poorly annotated and resist functional characterization by comparative approaches (partially due to their characteristically short length and atypical composition), several lines of evidence indicate that they encode functional proteins [20],[30], including structural *in vitro* analyses on *E. coli* ORFans (unpublished data). Therefore, the retention of ORFans may reside in the fact that they confer truly novel (but as yet unknown) functions, as opposed to traits that are apt to be redundant to the recipient organism. Alternatively, as ORFans are generally thought to be derived from selfish mobile elements (but see [28]), some might be perpetuated by encoding selfish functions themselves.

The distributions of ORFans and HOPs show that sequences that do not provide a useful function are eliminated and that bacterial genomes are not repositories of non-functional genes. This parallels the situation observed for pseudogenes, which, due to their rapid removal, are largely strain- or genome-specific [7]. Because the most-recently acquired genes are the least likely to supply an immediately useful function, we might expect that the newest genes in a genome are the most rapidly removed [18]. Indeed, comparing the two oldest branches indicates that while 33% of the genes gained on the ancestral branch are lost in each extant genome, 42% of the genes gained on a younger branch (SCE) are lost. From the present dataset, it is difficult to assess how this trend continues because relatively few genes are introduced on each branch (only 9 and 13 genes on SC and S4 respectively), and in younger clades, there are successively fewer genomes from which the gene can be eliminated. However, the low numbers of genes mapped to these internal branches probably reflects the fact that relatively few acquired genes are being maintained.

The density of sequenced genomes has allowed the use of phylogenetic methods to assess the dynamics of gene contents within several bacterial species, and has shown that rates of DNA gain and loss are often strain or lineage specific. Based on the same genomes analyzed in the present study, Hershberg and co-workers [26] found that the rates of gene loss in *Shigella* species were consistently higher than in related strains of *E. coli*, presumably due to reduced selection brought about by their small effective population sizes. Our data agree with these findings, and additionally, show that *Shigella* species also have lower rates of gene acquisition and lower rates of retaining acquired genes. Taken together, the inactivation and subsequent deletion of resident genes coupled with decreased levels of gene acquisition and subsequent persistence accounts for the reduced size of *Shigella* genomes.

Applying a similar approach, Vernikos and co-workers [31] analyzed the genes acquired by the strains of *Salmonella enterica* for which genome sequences are available. In *Salmonella*, most of the acquired genes have low GC-contents and are still “ameliorating”, *i.e.*, adjusting their base composition towards that of the host genome [31],[32], similar to results observed for acquired sequences in the Gammaproteobacteria as a whole [20]. That amelioration has been observed in studies on *Salmonella* and the Gammaproteobacteria, but not in *E. coli,* is due to the fact that the sequenced strains of *E. coli* span a much shorter timescale and have not yet accumulated sufficient numbers of mutations to noticeably alter the average base composition of genes.

In addition to assessing genome dynamics by following the presence and absence of acquired genes, we also traced the formation of pseudogenes to more closely monitor the mechanisms by which genes are inactivated and eliminated from these genomes. Pseudogenes in our analyses have restricted distributions, and nearly half of the inactivated ORFans and HOPs occur in only a single genome. In that the formation of pseudogenes is an ongoing process, their very restricted distributions denote that inactivated genes are eliminated rapidly from the genome and imply that newly acquired genes that are not immediately functional are also subject to rapid removal. Although such assessments of gene contents are based only on those genes now present in contemporary genomes, the recognition of pseudogenes can provide additional insights into the evolution and dynamics of genomes. The inclusion of pseudogenes in the present analysis provides some indication that high numbers of genes are gained and lost without leaving traces of their introgression [7],[33].

In conclusion, comparative genomics of multiple closely related strains provides high-resolution assessments and quantifications of gene fluxes in an evolutionary context [31],[34], and allows specific estimations of the processes of gene inactivation and deletion. Within the sequenced strains of *E. coli* and *Shigella* spp., we detected large differences among closely related lineages in the rates of gene acquisition and loss, but also differences in gene retention rates due to the source of acquired genes. The higher retention rate observed in the functionally obscure ORFan genes suggests that there are unknown adaptive benefits to these small acquired genes.

## Materials and Methods

### Strain Phylogeny

To trace the history of each gene in the sequenced *E. coli* and *Shigella* genomes, it is first necessary to resolve the phylogenetic relationships among these 13 strains. We based this phylogeny on the core set of single-copy genes identified by Lerat *et al*. [35] as showing virtually no evidence of lateral gene transfer within the Gammaproteobacteria. The seven sequenced *E. coli* genomes (*E. coli* K-12 [36], *E. coli* W3110, *E. coli* Sakai [37], *E. coli* EDL933 [38], *E. coli* CFT073 [21], *E. coli* UTI89 [39] and *E. coli* 536) and six sequenced *Shigella* genomes (*S. flexneri* 301 [40], *S. flexneri* 2457, *S. flexneri* 8401 [41], *S. dysenteriae* [22], *S. boydii* [22] and *S. sonnei* [22]) were searched via BLASTP [42] for orthologs of these core genes, applying an E-value<1^−10^ and a match length >75%. Of the 203 genes identified by Lerat *et al*. [35], 169 single copy genes met these criteria of orthology and were used for phylogenetic reconstruction. Concatenated sequences of these 169 genes from all 13 *E. coli* and *Shigella* genomes were aligned using MAFFT [43] and the alignment was edited to remove gaps using Gblocks [44]. A maximum likelihood tree (using DNAML module of PHYLIP; http://evolution.genetics.washington.edu/phylip.html) was generated using the concatenated orthologous sequences of *Salmonella enterica* as the outgroup.

### Identification of Acquired Genes

The genome sequences of the 367 prokaryotes (339 bacteria and 28 archaea) available at the time of this study were retrieved from GenBank (ftp.ncbi.nih.gov/genbank/genomes/Bacteria/; August 2006), and an in-house database was created by extracting protein sequences from all but the 13 *E. coli* and *Shigella* genomes.

Newly acquired genes can be of two types: ORFans, genes with no detectable homolog in the databases, and HOPs (heterogeneous occurrence in prokaryotes [20]), genes with homologs in distantly related species. ORFans in each of the 13 *E. coli* and *Shigella* genomes were identified as described previously in Daubin and Ochman [20]. In brief, all protein sequences from these genomes were compared with the database using BLASTP, applying an E-value cutoff of 0.01 to uncover distant homologs. Those genes without a match at this relaxed cutoff were considered to be potential ORFans. To eliminate possible artifacts due to annotation errors, we queried gammaproteobacterial genomes with all putative ORFans using TBLASTN and excluded those with matches having E-value cutoff<10^−5^ and alignment lengths >50%. The distribution of all remaining ORFans among the 13 strains of *E. coli* and *Shigella* were obtained by comparing the ORFans from each *E. coli* and *Shigella* genome with the remaining 12 genomes using TBLASTN with an E-value cutoff of 10^−5^. Based on their distribution among strains, ORFans were assigned to clades of the *E. coli* phylogeny. The orthology of ORFans present in more than one strain was confirmed by genome context.

In contrast to ORFans, HOPs have homologs in other prokaryotic genomes. To qualify as a HOP, a protein must be restricted to an *E. coli* clade, absent from closely related genomes, and have a homolog in a more distantly related prokaryotic genome. We performed BLAST analyses to identify genes that displayed such sporadic distributions. For example, the 9 HOPs restricted to clade S1 (Figure 1) were present in *S. flexneri* 301 and *S. flexneri* 2457, lacked homologs in the other *E. coli* and *Shigella* genomes, but had homologs in some distantly related genomes. We mapped the branch on which a gene was acquired by reconstructing the parsimonious scenario that explains the present-day gene distribution [18], such that the path that invokes the lowest number of events was viewed as the most evolutionarily plausible. In these reconstructions, gene gains and losses were viewed as individual and equally likely events. The genes acquired on each branch are listed in Supplementary Table S4. Classification of the identified ORFans and HOPs to Clusters of Orthologous Groups (COGs) [45] was performed using in-house scripts.

### Identification of Pseudogenes

Pseudogenes were identified by using Ψ-Φ as described previously [7],[9],[16]. In this procedure, the annotated proteins from each genome were queried against the complete nucleotide sequence of every other strain with E-value cutoffs of 10^−15^ and sequence identities >75%. The Ψ-Φ program suite uses the TBLASTN output to return lists of predicted disrupted genes, which are manually curated. To identify gene-inactivating mutations, the predicted pseudogenes were aligned against their orthologs using CLUSTALW [46]. Gene-inactivating mutations were grouped into five classes: frameshifts (insertions or deletions of 1 or 2 nucleotides in length), deletions (>2 nucleotides in length), insertions (>2 nucleotides in length), truncations (large deletions at either or both ends of a coding sequence), nonsense mutations, or a combination of different classes.

## Supporting Information

Figure S1 Base composition content of ORFans (A) and HOPs (B). Letters designate each of the branches in the phylogeny of *E. coli* and *Shigella* strains (see Figure 1). Values are depicted as means and standard errors.
(0.39 MB EPS)

Figure S2 The phylogenetic persistence of ORFans and HOPs in *E. coli* and *Shigella*. Gene gains (red), losses (purple) and pseudogenes (green); numbers above branches represent ORFans, those below, HOPs.
(0.43 MB EPS)

Figure S3 Percentages of intact (grey), disrupted (green) and deleted (purple) ORFans (A) and HOPs (B) in contemporary genomes.
(0.43 MB EPS)

Table S1 COG assignments of 652 HOPs.
(0.04 MB PDF)

Table S2 Rates of gain and loss of acquired genes.
(0.03 MB PDF)

Table S3 Mechanism of inactivation of ORFan and HOP pseudogenes.
(0.05 MB PDF)

Table S4 Point of acquisition of ORFans and HOPs.
(0.06 MB PDF)
