# Supplementary material for: The Emergence and Fate of Horizontally Acquired Genes in Escherichia coli
Source: PLoS Comput Biol. 2008 Apr 11;4(4):e1000059. doi: 10.1371/journal.pcbi.1000059 (PMC2275313; doi:10.1371/journal.pcbi.1000059)
Supplement: Table S1 — COG assignments of 652 HOPs. (0.04 MB PDF) [file pcbi.1000059.s004.pdf]

Supplementary Table S1. COG assignments of 652 HOPs.

COG Category	Percentage of HOPs
**Information storage and processing**	
[J] Translation, ribosomal structure and biogenesis	3
[A] RNA processing and modification	0
[K] Transcription	10
[L] Replication, recombination and repair	21
[B] Chromatin structure and dynamics	0
**sub total**	**34 (5.2%)**
**Cellular processes and signaling**	
[D] Cell cycle control, cell division, chromosome partitioning	1
[Y] Nuclear structure	0
[V] Defense mechanisms	4
[T] Signal transduction mechanisms	10
[M] Cell wall/membrane/envelope biogenesis	27
[N] Cell motility	0
[Z] Cytoskeleton	0
[W] Extracellular structures	0
[U] Intracellular trafficking, secretion, and vesicular transport	5
[O] Posttranslational modification, protein turnover, chaperones	7
**sub total**	**54 (8.3%)**
**Metabolism**	
[C] Energy production and conversion	10
[G] Carbohydrate transport and metabolism	10
[E] Amino acid transport and metabolism	11
[F] Nucleotide transport and metabolism	3
[H] Coenzyme transport and metabolism	3
[I] Lipid transport and metabolism	9
[P] Inorganic ion transport and metabolism	8
[Q] Secondary metabolites biosynthesis, transport and catabolism	9
**sub total**	**63 (9.7%)**
**Poorly Characterized**	
[R] General function prediction only	50
[S] Function unknown	50
no COG identified	401
**sub total**	**501 (76.8%)**
**Total**	**652 (100%)**
