# Supplementary material for: The Emergence and Fate of Horizontally Acquired Genes in Escherichia coli
Source: PLoS Comput Biol. 2008 Apr 11;4(4):e1000059. doi: 10.1371/journal.pcbi.1000059 (PMC2275313; doi:10.1371/journal.pcbi.1000059)
Supplement: Table S2 — Rates of gain and loss of acquired genes. (0.03 MB PDF) [file pcbi.1000059.s005.pdf]

Supplementary Table S2. Rates of gain and loss of acquired genes in each lineage

	Branch	Branch length	Gains (kb)	Gain Rate* (kb/0.01)	Losses (kb)	Loss Rate* (kb/0.01)
**Clade**	SCE	0.00408	118	289	1.4	3
	SC	0.00185	6.1	33	18	97
	S4	0.00138	4.8	35	31	225
	S3	0.00145	0	0	51	352
	S2	0.00498	21	42	76	153
	S1	0.00039	5.9	151	29	744
	C1	0.00427	23	54	55	129
	E1	0.00406	132	325	19	47
	E2	0.00232	28	121	23	99
	U1	0.00136	26	191	6.3	46
	U2	0.00642	117	182	11	17
**Strain**	*S. flexneri* 301	0.00019	5	263	5	263
	S. flexneri 2457	0.00021	0.5	24	18	857
	S. flexneri 8401	0.00044	4.4	100	52	1182
	S. boydii	0.00679	23	34	65	96
	S. sonnei	0.00340	21	62	49	144
	S. dysenteriae	0.00565	8.6	15	163	288
	E. coli K-12	0.00006	0	0	NA	NA
	E. coli W3110	0.00008	0	0	NA	NA
	E. coli EDL933	0.00018	4.5	250	11	1833
	E. coli Sakai	0.00006	20	3333	19	1056
	E. coli UTI89	0.00288	47	163	30	104
	E. coli CFT073	0.00315	55	175	47	149
	E. coli 536	0.00252	22	87	33	131

** The gain (loss) rate is calculated as the amount of DNA gained (lost) in kilobases for a branch length of 0.01.*
