# Supplementary material for: The Emergence and Fate of Horizontally Acquired Genes in Escherichia coli
Source: PLoS Comput Biol. 2008 Apr 11;4(4):e1000059. doi: 10.1371/journal.pcbi.1000059 (PMC2275313; doi:10.1371/journal.pcbi.1000059)
Supplement: Table S3 — Mechanism of inactivation of ORFan and HOP pseudogenes. (0.05 MB PDF) [file pcbi.1000059.s006.pdf]

Supplementary Table S3. Mechanisms of inactivation of ORFans and HOPs

	ORFans*								HOPs*							
	**Total**	**F**	**IS**	**SC**	**T**	**>=2**	**D**	**I**	**Total**	**F**	**IS**	**SC**	**T**	**>=2**	**D**	**I**
S. flexneri 301	60	5	5	14	27	8	0	1	26	1	8	4	8	5	0	0
S. flexneri 2457	70	8	7	18	30	6	0	1	32	2	12	7	6	5	0	0
S. flexneri 8401	91	20	6	29	26	7	2	1	22	2	7	6	5	2	0	0
S. boydii	55	4	10	7	29	4	0	1	17	1	10	2	3	1	0	0
S. sonnei	47	4	10	7	21	5	0	0	28	2	13	1	5	4	3	0
S. dysenteriae	62	2	22	4	29	3	2	0	16	2	8	2	1	3	0	0
E. coli K-12	39	2	3	8	19	7	0	0	9	0	0	1	5	3	0	0
E. coli W3110	37	1	1	9	20	6	0	0	9	0	0	1	5	3	0	0
E. coli EDL933	33	3	2	4	19	5	0	0	1	0	0	0	0	1	0	0
E. coli Sakai	27	1	0	4	18	4	0	0	3	0	0	0	2	1	0	0
E. coli UTI89	28	2	0	4	16	6	0	0	2	1	0	0	1	0	0	0
E. coli CFT073	37	4	2	6	21	4	0	0	10	6	0	1	2	0	1	0
E. coli 536	50	11	0	11	16	9	2	1	8	0	0	1	3	2	1	1

*Abbreviations are as follows:

F: Frameshift

IS: Insertion sequence

SC: Stop codon

T: Truncation

>=2: More than 1 type of mutation

D: Deletion

I: Insertion
