# Supplementary material for: The Emergence and Fate of Horizontally Acquired Genes in Escherichia coli
Source: PLoS Comput Biol. 2008 Apr 11;4(4):e1000059. doi: 10.1371/journal.pcbi.1000059 (PMC2275313; doi:10.1371/journal.pcbi.1000059)
Supplement: Table S4 — Point of acquisition of ORFans and HOPs. (0.06 MB PDF) [file pcbi.1000059.s007.pdf]

ORFans
Ancestral_ORFans
NP_751964.1
NP_751970.1
NP_751992.1
NP_751996.1
NP_752045.1
NP_752047.1
NP_752074.1
NP_752111.1
NP_752132.1
NP_752134.1
NP_752216.1
NP_752221.1
NP_752229.1
NP_752232.1
NP_752375.1
NP_752381.1
NP_752405.1
NP_752415.1
NP_752419.1
NP_752422.1
NP_752431.1
NP_752434.1
NP_752441.1
NP_752481.1
NP_752483.1
NP_752501.1
NP_752517.1
NP_752601.1
NP_752628.1
NP_752686.1
NP_752708.1
NP_752769.1
NP_752811.1
NP_752817.1
NP_752827.1
NP_752938.1
NP_752940.1
NP_752943.1
NP_752964.1
NP_752980.1
NP_753019.1
NP_753037.1
NP_753051.1
NP_753054.1
NP_753067.1
NP_753083.1
NP_753206.1
NP_753207.1
NP_753216.1
NP_753217.1
NP_753225.1

NP_753228.1
NP_753230.1
NP_753248.1
NP_753277.1
NP_753278.1
NP_753285.1
NP_753312.1
NP_753315.1
NP_753331.1
NP_753333.1
NP_753340.1
NP_753341.1
NP_753352.1
NP_753366.1
NP_753382.1
NP_753427.1
NP_753444.1
NP_753494.1
NP_753523.1
NP_753525.1
NP_753529.1
NP_753541.1
NP_753558.1
NP_753560.1
NP_753588.1
NP_753607.1
NP_753648.1
NP_753656.1
NP_753666.1
NP_753667.1
NP_753685.1
NP_753739.1
NP_753805.1
NP_753812.1
NP_753825.1
NP_753875.1
NP_753884.1
NP_753936.1
NP_753941.1
NP_753942.1
NP_753956.1
NP_753965.1
NP_754013.1
NP_754016.1
NP_754024.1
NP_754043.1
NP_754063.1
NP_754095.1
NP_754097.1
NP_754114.1
NP_754151.1
NP_754158.1
NP_754180.1

NP_754192.1
NP_754209.1
NP_754210.1
NP_754220.1
NP_754243.1
NP_754260.1
NP_754263.1
NP_754267.1
NP_754330.1
NP_754334.1
NP_754373.1
NP_754393.1
NP_754422.1
NP_754428.1
NP_754462.1
NP_754500.1
NP_754512.1
NP_754533.1
NP_754534.1
NP_754535.1
NP_754536.1
NP_754550.1
NP_754554.1
NP_754569.1
NP_754574.1
NP_754591.1
NP_754613.1
NP_754614.1
NP_754628.1
NP_754634.1
NP_754635.1
NP_754641.1
NP_754671.1
NP_754700.1
NP_754723.1
NP_754742.1
NP_754773.1
NP_754785.1
NP_754795.1
NP_754800.1
NP_754845.1
NP_754874.1
NP_754875.1
NP_754898.1
NP_754904.1
NP_754927.1
NP_754928.1
NP_754965.1
NP_754994.1
NP_755057.1
NP_755096.1
NP_755101.1
NP_755102.1

NP_755115.1
NP_755130.1
NP_755217.1
NP_755219.1
NP_755232.1
NP_755239.1
NP_755246.1
NP_755341.1
NP_755361.1
NP_755362.1
NP_755394.1
NP_755401.1
NP_755420.1
NP_755505.1
NP_755536.1
NP_755547.1
NP_755550.1
NP_755682.1
NP_755697.1
NP_755700.1
NP_755702.1
NP_755706.1
NP_755712.1
NP_755745.1
NP_755815.1
NP_755838.1
NP_755870.1
NP_755876.1
NP_755891.1
NP_756032.1
NP_756038.1
NP_756039.1
NP_756046.1
NP_756059.1
NP_756094.1
NP_756105.1
NP_756111.1
NP_756114.1
NP_756150.1
NP_756162.1
NP_756164.1
NP_756165.1
NP_756167.1
NP_756171.1
NP_756172.1
NP_756173.1
NP_756174.1
NP_756200.1
NP_756214.1
NP_756215.1
NP_756225.1
NP_756233.1
NP_756236.1

NP_756253.1
NP_756282.1
NP_756297.1
NP_756299.1
NP_756334.1
NP_756432.1
NP_756439.1
NP_756444.1
NP_756466.1
NP_756471.1
NP_756483.1
NP_756503.1
NP_756505.1
NP_756546.1
NP_756562.1
NP_756579.1
NP_756639.1
NP_756662.1
NP_756665.1
NP_756666.1
NP_756668.1
NP_756700.1
NP_756714.1
NP_756757.1
NP_756814.1
NP_756819.1
NP_756825.1
NP_756850.1
NP_756851.1
NP_756852.1
NP_756860.1
NP_756896.1
NP_756903.1
NP_756907.1
NP_756917.1
NP_756927.1
NP_756977.1
NP_756980.1
NP_756983.1
NP_756985.1
NP_757077.1
NP_757169.1
NP_757171.1
NP_757190.1
NP_757246.1
NP_757259.1
NP_757262.1
NP_757294.1
NP_757310.1
NP_753314.1
YP_539866.1
YP_541279.1
YP_541470.1

AP_004237.1
AP_001227.1
AP_001187.1
AP_000980.1
AP_002197.1
AP_002602.1
AP_004629.1
AP_000949.1
AP_001447.1
AP_001621.1
AP_001797.1
YP_409336.1
YP_688988.1
YP_689024.1
NP_837744.1
NP_838529.1
NP_836713.1
NP_705967.1
NP_706060.1
NP_707647.1
NP_707900.1
NP_708861.1
NP_708157.1
YP_401827.1
YP_401749.1
YP_401973.1
YP_402005.1
YP_402304.1
YP_402767.1
YP_403379.1
YP_403434.1
YP_403744.1
YP_403753.1
YP_403756.1
YP_403757.1
YP_403977.1
YP_404133.1
YP_405107.1
YP_405602.1
YP_405935.1
YP_405219.1
NP_309640.1
BAB35084.1
NP_309700.1
NP_309864.1
BAB35362.1
NP_310917.1
BAB36907.1
BAB37214.1
NP_311961.1
NP_288149.1
NP_285693.1
NP_287934.1

NP_288626.1
NP_286076.1
NP_286308.1
NP_286336.1
NP_287023.1
NP_287631.1
NP_288050.1
NP_288330.1
NP_288716.1
NP_288961.1
NP_289174.1
NP_289213.1
NP_289275.1
NP_289283.1
NP_289320.1
NP_289590.1
NP_289694.1
NP_289822.1
NP_289958.1
NP_290081.1
NP_290159.1
YP_667971.1
YP_668269.1
YP_668310.1
YP_668322.1
YP_668335.1
YP_668379.1
YP_668654.1
YP_668748.1
YP_669021.1
YP_669125.1
YP_669233.1
YP_669334.1
YP_669510.1
YP_669554.1
YP_669673.1
YP_669745.1
YP_669801.1
YP_669854.1
YP_669893.1
YP_669946.1
YP_670060.1
YP_670295.1
YP_670373.1
YP_670532.1
YP_670564.1
YP_670821.1
YP_670898.1
YP_671243.1
YP_671729.1
YP_671732.1
YP_671859.1
YP_672099.1

YP_672223.1
YP_672252.1
YP_672485.1
YP_672494.1
YP_310027.1
YP_313014.1
YP_539157.1
YP_539346.1
YP_539347.1
YP_539360.1
YP_539367.1
YP_539413.1
YP_539443.1
YP_539616.1
YP_539724.1
YP_539799.1
YP_539835.1
YP_540120.1
YP_540428.1
YP_540454.1
YP_540676.1
YP_540776.1
YP_540826.1
YP_540836.1
YP_540868.1
YP_540918.1
YP_540942.1
YP_540999.1
YP_541013.1
YP_541033.1
YP_541034.1
YP_541114.1
YP_541202.1
YP_541315.1
YP_541372.1
YP_541433.1
YP_541471.1
YP_541572.1
YP_541869.1
YP_541877.1
YP_541949.1
YP_542668.1
YP_542669.1
YP_542959.1
YP_542966.1
YP_543018.1
YP_543200.1
YP_543543.1
YP_543611.1
YP_543803.1
NP_752050.1
NP_756183.1
NP_755001.1

NP_755744.1
NP_756488.1
NP_752338.1
NP_753317.1
NP_753316.1
NP_753343.1
NP_753961.1
NP_754420.1
NP_754501.1
NP_755653.1
NP_755708.1
NP_752435.1
NP_753521.1
NP_754794.1
NP_755618.1
YP_407073.1
YP_671960.1
YP_671468.1
AP_002044.1
YP_309675.1
NP_706055.1
YP_542142.1
NP_309419.1
YP_671691.1
YP_408355.1
YP_404330.1
YP_691047.1
YP_669266.1
YP_672141.1
AP_003992.1
YP_672196.1
YP_672493.1
YP_402511.1
YP_670078.1
NP_290835.1
NP_286307.1
YP_669723.1
YP_407475.1
NP_309760.1
NP_706509.1
NP_289692.1
YP_309668.1
YP_403263.1
NP_309733.1
YP_670894.1
NP_757238.1
NP_754012.1

SCE_ORFans
YP_409327.1
YP_410448.1
YP_690862.1
YP_691259.1

YP_690523.1
YP_690834.1
YP_691148.1
NP_838195.1
NP_839634.1
NP_709283.1
NP_709865.1
NP_706944.1
NP_707671.2
NP_709872.1
NP_309801.1
NP_287685.1
NP_289205.1
NP_289718.1
NP_290681.1
YP_310877.1
AP_001193.1
AP_000720.1
AP_000721.1
AP_000979.1
AP_001632.1
AP_002093.1
AP_002349.1
AP_002548.1
AP_002945.1
AP_003229.1
AP_003413.1
AP_003418.1
AP_003423.1
AP_003424.1
AP_004304.1
AP_004303.1
AP_004115.1
AP_004548.1
AP_004704.1
AP_004817.1
AP_001021.1
AP_002078.1
AP_002148.1
AP_003227.1
AP_003228.1
AP_003231.1
AP_004715.1
YP_402912.1
NP_309389.1
NP_312214.1
YP_309055.1
NP_290183.1
NP_286637.1
NP_309603.1
NP_836519.1
NP_308978.1
BAB34786.1

YP_410344.1
NP_290515.1
NP_311891.1
NP_309270.1
NP_309115.1
YP_310820.1

E1_ORFans
NP_308262.1
NP_308266.1
NP_308267.1
NP_308299.1
BAB33709.1
BAB33731.1
BAB33973.1
BAB34027.1
NP_308631.1
NP_308831.1
BAB34240.1
NP_308872.1
NP_308874.1
NP_308876.1
NP_308941.1
BAB34479.1
NP_309088.1
BAB34485.1
NP_309090.1
BAB34489.1
NP_309097.1
NP_309099.1
BAB34501.1
NP_309121.1
NP_309125.1
NP_309124.1
NP_309126.1
NP_309154.1
NP_309189.1
NP_309192.1
NP_309195.1
NP_309219.1
NP_309220.1
NP_309245.2
NP_309257.1
NP_309258.1
NP_309261.1
NP_309262.1
BAB34729.1
NP_309342.1
BAB34741.1
NP_309347.1
BAB34752.1
BAB34754.1
NP_309361.1

NP_309384.1
BAB34784.1
BAB34808.1
NP_309543.1
NP_309544.1
NP_309553.1
BAB34985.1
BAB34986.1
NP_309596.1
NP_309605.1
NP_309607.1
NP_309612.1
BAB35023.1
NP_309790.1
BAB35233.1
NP_309839.1
NP_309976.1
NP_309979.1
NP_309987.1
NP_310009.1
NP_310169.1
BAB35581.1
NP_310238.1
NP_310239.1
NP_310254.1
NP_310255.1
NP_310276.1
NP_310280.1
NP_310290.1
BAB35689.1
NP_310298.1
NP_310309.1
NP_310310.1
BAB36044.1
BAB36071.1
NP_310741.1
BAB36170.1
NP_310785.1
NP_310786.1
NP_310787.1
NP_310792.1
BAB36257.1
NP_311006.1
NP_311018.1
BAB36416.1
BAB36431.1
BAB36660.1
NP_311526.1
BAB37268.1
BAB37274.1
NP_311881.1
NP_312575.1
NP_312593.1

BAB38012.1
NP_312619.1
NP_312983.1
NP_313274.1
NP_313294.1
NP_313318.1
NP_313319.1
NP_288545.1
NP_285712.1
NP_285905.1
NP_285926.1
NP_285995.1
NP_285996.1
NP_286021.1
NP_286022.1
NP_286290.1
NP_286418.1
NP_286673.1
NP_286676.1
NP_286689.1
NP_286690.1
NP_286691.1
NP_286741.1
NP_287022.1
NP_287036.1
NP_287043.1
NP_287044.1
NP_287045.1
NP_287055.1
NP_287057.1
NP_287060.1
NP_287699.1
NP_287700.1
NP_287762.1
NP_287944.1
NP_287949.1
NP_288358.1
NP_288608.1
NP_288609.1
NP_288610.1
NP_288926.1
NP_288927.1
NP_288929.1
NP_289177.1
NP_289194.1
NP_289416.1
NP_289425.1
NP_289545.1
NP_289552.1
NP_289554.1
NP_289991.1
NP_290262.1
NP_290263.1

NP_290264.1
NP_290266.1
NP_290270.1
NP_290274.1
NP_290278.1
NP_290285.1
NP_290287.1
NP_290289.1
NP_290306.1
NP_290307.1
NP_290342.1
NP_290353.1
NP_290367.1
NP_290571.1
NP_290903.1
NP_290907.1
NP_290909.1
NP_290911.1
NP_290923.1
NP_287951.1
NP_287952.1
NP_287959.1
NP_753114.1
NP_756379.1
NP_757038.1
NP_757054.1
YP_687843.1
YP_668263.1
YP_539555.1
YP_539929.1
YP_539930.1
YP_539976.1
YP_539977.1
YP_541693.1
YP_544068.1

E2_ORFans
YP_401954.1
YP_402021.1
YP_402023.1
YP_402052.1
YP_402474.1
YP_403256.1
YP_405553.1
YP_405587.1
BAB33443.1
NP_309232.1
NP_309238.2
NP_309427.1
NP_310998.1
NP_311000.1
NP_312683.1
BAB38081.1

NP_312686.1
NP_312892.1
NP_285765.1
NP_285906.1
NP_285907.1
NP_285908.1
NP_286457.1
NP_286458.1
NP_286459.1
NP_286460.1
NP_286561.1
NP_287021.1
NP_287749.1
NP_287750.1
NP_287751.1
NP_288522.1
NP_289403.1
NP_289960.1
NP_289970.1
NP_290308.1
NP_290340.1
NP_290354.1
NP_290558.1
NP_290572.1
NP_309275.1
NP_309564.1
NP_290305.1
NP_286142.1
YP_408344.1
YP_408347.1
YP_539554.1
YP_539557.1

S1_ORFans
NP_708644.2
NP_707863.1
NP_838475.1
YP_177613.1
NP_707479.2
NP_707481.1
NP_707480.2
YP_177614.1
AAN82069.1
AAN79751.1
AAN79750.1
AAN82068.1

S2_ORFans
NP_838991.1
YP_688676.1
NP_706166.1
NP_707044.1
NP_708293.1

NP_708292.1
NP_709258.1
NP_708290.1
NP_838007.1
NP_708294.2
NP_707925.1
NP_708778.1
NP_708291.1
NP_706527.1
NP_707102.1
NP_706217.1
YP_691179.1
NP_706216.1
NP_709261.1
NP_707506.1
NP_836749.1
NP_837650.1
AAN80843.1
NP_706794.1
NP_706593.1

S4_ORFans
NP_838125.1
NP_707687.1
NP_837602.1
NP_836747.1
NP_707455.1
SBO_1928
AAN79959.1
AAN79887.1
AAN79961.1

SC_ORFans
BAE76391.1
NP_708751.1
NP_708750.1

C1_ORFans
BAE76358.1
BAE76701.1
BAE76060.1
BAE76702.1
BAE76378.1
BAE76704.1
BAE76758.1
BAE76473.1
BAE76064.1
BAE76682.1
BAA35360.1
BAE76419.1
BAE76467.1
BAE76683.1
BAE76771.1

AAN81595.1
AAG55022.1
AAN81635.1

U1_ORFans
AAN79645.1
AAN83268.1
AAN80069.1
ABE06779.1
ABE07198.1
AAN78845.1
AAN79996.1
ABE06617.1
ABE06767.1
AAN79904.1
AAN80014.1
ABE06973.1
AAN81651.1

U2_ORFans
YP_540133.1
NP_751965.1
NP_751982.1
NP_752029.1
NP_752032.1
NP_752087.1
NP_752088.1
NP_752123.1
NP_752124.1
NP_752242.1
NP_752243.1
NP_752244.1
NP_752245.1
NP_752246.1
NP_752247.1
NP_752248.1
NP_752256.1
NP_752273.1
NP_752305.1
NP_752331.1
NP_752332.1
NP_752335.1
NP_752571.1
NP_752579.1
NP_752738.1
NP_752739.1
NP_752740.1
NP_753138.1
NP_753139.1
NP_753163.1
NP_753169.1
NP_753379.1
NP_753380.1

NP_753431.1
NP_753475.1
NP_753476.1
NP_753501.1
NP_753504.1
NP_753565.1
NP_753734.1
NP_753779.1
NP_753787.1
NP_753788.1
NP_753789.1
NP_753790.1
NP_754187.1
NP_754295.1
NP_754299.1
NP_754301.1
NP_754307.1
NP_754354.1
NP_754357.1
NP_754367.1
NP_754390.1
NP_754391.1
NP_754395.1
NP_754401.1
NP_754411.1
NP_754412.1
NP_754453.1
NP_754615.1
NP_754945.1
NP_754946.1
NP_755182.1
NP_755214.1
NP_755279.1
NP_755285.1
NP_755446.1
NP_755447.1
NP_755560.1
NP_755885.1
NP_756068.1
NP_756069.1
NP_756075.1
NP_756284.1
NP_756413.1
NP_756415.1
NP_756419.1
NP_756425.1
NP_756428.1
NP_756441.1
NP_756454.1
NP_756611.1
NP_756615.1
NP_756625.1
NP_756629.1

NP_756829.1
NP_756885.1
NP_756894.1
NP_756895.1
NP_757007.1
NP_757017.1
NP_757019.1
NP_757031.1
NP_757042.1
NP_757047.1
NP_757048.1
NP_757199.1
NP_757280.1
NP_755458.1
NP_755468.1
NP_755463.1
NP_753151.1
YP_539293.1
YP_539331.1
YP_539544.1
YP_539545.1
YP_539819.1
YP_540324.1
YP_540411.1
YP_540716.1
YP_541207.1
YP_541245.1
YP_541258.1
YP_541261.1
YP_541264.1
YP_541905.1
YP_541906.1
YP_541912.1
YP_542087.1
YP_543840.1
YP_543841.1
YP_543854.1
YP_543855.1
YP_543857.1
YP_543858.1
YP_543866.1
YP_543868.1
YP_543879.1
YP_543891.1
YP_543892.1
YP_543895.1
YP_543901.1
YP_543916.1
YP_543927.1
YP_543928.1
YP_668056.1
YP_668214.1
YP_668216.1

YP_668223.1
YP_668246.1
YP_668279.1
YP_669108.1
YP_669367.1
YP_669371.1
YP_669818.1
YP_669880.1
YP_669887.1
YP_669916.1
YP_669918.1
YP_669927.1
YP_669929.1
YP_669930.1
YP_669932.1
YP_670864.1
YP_670887.1
YP_670888.1
YP_671651.1
YP_671653.1
YP_671658.1
YP_671664.1
YP_671665.1
YP_671666.1
YP_672291.1
YP_672364.1
YP_672365.1
YP_672367.1
YP_672369.1
YP_672380.1
YP_672394.1
YP_672395.1
YP_672396.1
YP_672419.1
YP_672450.1
YP_672460.1
YP_670886.1
YP_541689.1
YP_312447.1

Singleton_ORFans
YP_539327.1
YP_539960.1
YP_539971.1
YP_539973.1
YP_539974.1
YP_539982.1
YP_540486.1
YP_540747.1
YP_541650.1
YP_541662.1
YP_541674.1
YP_541678.1

YP_541982.1
YP_541986.1
YP_542007.1
YP_542188.1
YP_543876.1
YP_543957.1
YP_543990.1
YP_544047.1
YP_544061.1
YP_544093.1
NP_752199.1
NP_752223.1
NP_752224.1
NP_752225.1
NP_752226.1
NP_752227.1
NP_752236.1
NP_752237.1
NP_752258.1
NP_752264.1
NP_752268.1
NP_752282.1
NP_752285.1
NP_752287.1
NP_752290.1
NP_752295.1
NP_752296.1
NP_752874.1
NP_753103.1
NP_753129.1
NP_753131.1
NP_753132.1
NP_753134.1
NP_753268.1
NP_753324.1
NP_753325.1
NP_753326.1
NP_753328.1
NP_753329.1
NP_753394.1
NP_753395.1
NP_753397.1
NP_753407.1
NP_753416.1
NP_753434.1
NP_753435.1
NP_753437.1
NP_753987.1
NP_754059.1
NP_755088.1
NP_755123.1
NP_755150.1
NP_755151.1

NP_755269.1
NP_755433.1
NP_755473.1
NP_755477.1
NP_755478.1
NP_755479.1
NP_755480.1
NP_755481.1
NP_755506.1
NP_755507.1
NP_755528.1
NP_755567.1
NP_756041.1
NP_756391.1
NP_756402.1
NP_756403.1
NP_756404.1
NP_756414.1
NP_756420.1
NP_756421.1
NP_756422.1
NP_756423.1
NP_757011.1
NP_757012.1
NP_757013.1
NP_757021.1
NP_757058.1
NP_757060.1
NP_757222.1
NP_757223.1
NP_757224.1
NP_757228.1
YP_407081.1
YP_407835.1
YP_408029.1
YP_408524.1
YP_408532.1
YP_409956.1
YP_410035.1
YP_410038.1
YP_410427.1
YP_687847.1
YP_689357.1
YP_689499.1
YP_402673.1
YP_403412.1
YP_403526.1
YP_404755.1
YP_404911.1
NP_309207.1
NP_309210.1
NP_309218.1
BAB34621.1

NP_312974.1
NP_312975.1
NP_312977.1
NP_312978.1
NP_312980.1
NP_312982.1
NP_312984.1
NP_312986.1
NP_313023.1
YP_668256.1
YP_669230.1
YP_670589.1
YP_670590.1
YP_670702.1
YP_670871.1
YP_670914.1
YP_670916.1
YP_670917.1
YP_671641.1
YP_671642.1
YP_671669.1
YP_671674.1
YP_671680.1
YP_671681.1
YP_671685.1
YP_671688.1
YP_671697.1
YP_671711.1
YP_671718.1
YP_310239.1
YP_310825.1
YP_310826.1
YP_310828.1
YP_311532.1
YP_311514.1

HOPs

Ancestral_HOPs
YP_541676.1
YP_541965.1
YP_541969.1
YP_541970.1
YP_541971.1
YP_541973.1
YP_541976.1
YP_541977.1
YP_541981.1
YP_542041.1
YP_542122.1
YP_542374.1
YP_543675.1
NP_754652.1
NP_752215.1

NP_752222.1
NP_752284.1
NP_752294.1
NP_752404.1
NP_753095.1
NP_753122.1
NP_753135.1
NP_753192.1
NP_753198.1
NP_754304.1
NP_754378.1
NP_754385.1
NP_754410.1
NP_754413.1
NP_754414.1
NP_755122.1
NP_755124.1
NP_755216.1
NP_755218.1
NP_755319.1
NP_755321.1
NP_755334.1
NP_755373.1
NP_755424.1
NP_755455.1
NP_755476.1
NP_755527.1
NP_755543.1
NP_755544.1
NP_755549.1
NP_755590.1
NP_756361.1
NP_756383.1
NP_756426.1
NP_756430.1
NP_756431.1
NP_756594.1
NP_756595.1
NP_757003.1
NP_757004.1
NP_757008.1
NP_757046.1
NP_756331.1
NP_755705.1
NP_756190.1
NP_753818.1
NP_752340.1
NP_752376.1
NP_752508.1
NP_752584.1
NP_752776.1
NP_752819.1
NP_753684.1

NP_753692.1
NP_753746.1
NP_753751.1
NP_753792.1
NP_753955.1
NP_753962.1
NP_754485.1
NP_754517.1
NP_754537.1
NP_754538.1
NP_754539.1
NP_754540.1
NP_754658.1
NP_754654.1
NP_754655.1
NP_754657.1
NP_754701.1
NP_754702.1
NP_755084.1
NP_755220.1
NP_755320.1
NP_755333.1
NP_755336.1
NP_755592.1
NP_755593.1
NP_755709.1
NP_755958.1
NP_756151.1
NP_756277.1
NP_756449.1
NP_756479.1
NP_756472.1
NP_756981.1
NP_756982.1
NP_757263.1
NP_757264.1
NP_754953.1
NP_755328.1
NP_755329.1
NP_755772.1
YP_668180.1
YP_669022.1
YP_670012.1
YP_670755.1
YP_670895.1
YP_670940.1
YP_671723.1
YP_672467.1
NP_285931.1
NP_286289.1
NP_286420.1
NP_287697.1
NP_288089.1

SCE_HOPs
NP_290072.1
NP_286081.1
NP_285943.1
NP_288847.1
NP_290526.1
NP_289094.1
NP_289095.1
NP_289096.1
NP_289038.1
NP_286091.1
NP_286094.1
NP_290173.1
NP_286413.1
NP_290205.1
NP_287696.1
NP_288238.1
NP_288393.1
NP_289303.1
NP_289310.1
NP_289405.1
NP_289406.1
NP_289485.1
NP_290174.1
NP_290175.1
NP_290506.1
NP_290699.1
NP_290959.1
NP_287637.1
NP_289400.1
NP_289401.1
NP_285929.1
NP_285942.1
NP_286131.1
NP_286246.1
NP_286291.1
NP_286292.1
NP_286293.1
NP_287636.1
NP_287667.1
NP_287702.1
NP_287850.1
NP_288195.1
NP_288395.1
NP_288969.1
NP_289207.1
NP_289305.1
NP_289306.1
NP_289307.1
NP_289308.1
NP_289309.1
NP_289433.1

NP_289971.1
NP_290007.1
NP_290055.1
NP_290162.1
NP_290568.1
YP_402505.1
YP_402718.1
YP_403587.1
YP_404287.1
YP_405647.1
YP_403787.1
YP_403786.1
YP_405236.1
YP_402318.1
YP_402406.1
YP_403334.1
YP_403523.1
YP_403549.1
YP_403701.1
YP_403980.1
YP_405038.1

SC_HOPs
NP_415805.1
NP_418261.1
NP_415216.2
NP_414753.1
NP_414706.2
NP_415260.1

S1_HOPs
NP_706163.1
NP_706261.1
NP_707926.2
NP_708782.1

S2_HOPs
YP_689523.1
YP_687785.1
YP_687787.1
YP_687788.1
YP_687786.1
YP_687800.1
YP_687796.1
YP_687892.1
YP_687969.1
YP_687970.1
YP_689355.1
YP_689378.1
YP_690844.1

S4_HOPs
YP_309706.1

YP_312040.2
YP_313206.1
YP_313207.1

C1_HOPs
NP_417494.1
NP_416540.1
NP_418766.4
NP_418765.1
NP_415906.1
NP_415907.1
NP_415908.1
NP_415909.3
NP_415914.1
NP_415917.1
NP_418080.1
NP_418081.1
NP_415904.1
NP_416538.1
NP_415216.1
NP_416575.1
NP_416776.1
NP_417239.1
NP_417493.1
YP_588477.1
NP_415954.2

E1_HOPs
NP_290258.1
NP_290252.1
NP_290273.1
NP_290256.1
NP_290255.1
NP_290250.1
NP_290257.1
NP_286707.1
NP_287115.1
NP_286708.1
NP_287116.1
NP_286710.1
NP_287118.1
NP_286711.1
NP_287119.1
NP_286712.1
NP_287120.1
NP_286699.1
NP_287107.1
NP_286706.1
NP_287114.1
NP_286678.1
NP_287086.1
NP_286679.1
NP_287087.1

NP_286680.1
NP_287088.1
NP_286677.1
NP_287085.1
NP_286681.1
NP_287089.1
NP_286682.1
NP_287090.1
NP_288541.1
NP_286088.1
NP_286451.1
NP_286671.1
NP_286701.1
NP_286703.1
NP_286704.1
NP_286705.1
NP_286736.1
NP_287039.1
NP_287059.1
NP_287079.1
NP_287109.1
NP_287111.1
NP_287112.1
NP_287113.1
NP_287144.1
NP_287316.1
NP_287604.1
NP_287605.1
NP_287945.1
NP_288200.1
NP_288607.1
NP_288721.1
NP_288928.1
NP_289176.1
NP_289178.1
NP_289192.1
NP_289195.1
NP_289196.1
NP_289197.1
NP_289198.1
NP_289199.1
NP_289413.1
NP_289414.1
NP_289415.1
NP_289419.1
NP_289423.1
NP_289426.1
NP_289429.1
NP_289544.1
NP_289553.1
NP_289557.1
NP_289558.1
NP_289990.1

NP_290056.1
NP_290260.1
NP_290450.1
NP_290904.1
NP_290915.1
NP_290917.1
NP_290920.1
NP_290921.1
NP_290970.1

E2_HOPs
YP_402349.1
YP_402350.1
YP_402351.1
YP_402352.1
YP_403521.1
YP_403828.1
YP_403829.1
YP_404453.1
YP_405064.1
YP_405114.1
YP_405543.1

U1_HOPs
YP_539056.1
YP_539225.1
YP_539457.1
YP_539499.1
YP_539840.1
YP_540057.1
YP_540086.1
YP_540116.1
YP_540281.1
YP_540302.1
YP_540312.1
YP_540734.1
YP_541668.1
YP_541940.1
YP_541989.1
YP_542182.1
YP_542262.1
YP_542345.1
YP_542438.1
YP_542583.1
YP_542727.1
YP_542730.1
YP_542741.1
YP_542744.1
YP_542752.1
YP_542811.1
YP_542842.1
YP_543123.1
YP_543503.1

YP_543628.1
YP_544008.1
YP_544058.1

U2_HOPs
YP_667960.1
YP_668005.1
YP_668053.1
YP_668055.1
YP_668057.1
YP_668177.1
YP_668182.1
YP_668183.1
YP_668221.1
YP_668226.1
YP_668248.1
YP_668474.1
YP_668481.1
YP_668620.1
YP_669387.1
YP_669814.1
YP_669815.1
YP_669816.1
YP_669819.1
YP_669821.1
YP_669825.1
YP_669826.1
YP_669828.1
YP_669844.1
YP_669845.1
YP_669846.1
YP_669863.1
YP_669864.1
YP_669869.1
YP_669876.1
YP_669905.1
YP_669975.1
YP_669977.1
YP_669978.1
YP_670021.1
YP_670022.1
YP_670023.1
YP_670024.1
YP_670318.1
YP_670403.1
YP_670610.1
YP_670689.1
YP_670776.1
YP_670865.1
YP_670905.1
YP_670911.1
YP_670912.1
YP_671014.1

YP_671556.1
YP_671735.1
YP_671885.1
YP_671886.1
YP_671894.1
YP_671902.1
YP_671903.1
YP_671904.1
YP_671958.1
YP_672010.1
YP_672081.1
YP_672293.1
YP_672294.1
YP_672342.1
YP_672393.1
YP_672408.1
YP_672411.1
YP_672412.1
YP_672413.1
YP_672458.1
YP_672477.1
YP_672558.1

Singleton_HOPs
YP_312670.1
YP_311520.1
YP_311521.1
YP_310951.1
YP_310952.1
YP_310953.1
YP_310954.1
YP_310955.1
YP_310959.1
YP_310241.1
YP_310548.1
YP_311519.1
YP_311533.1
YP_312665.1
YP_312666.1
YP_312667.1
YP_312668.1
YP_312669.1
YP_407149.1
YP_407255.1
YP_407402.1
YP_407601.1
YP_407602.1
YP_407603.1
YP_407605.1
YP_407679.1
YP_408536.1
YP_410036.1
YP_410416.1

YP_410422.1
YP_410428.1
YP_410599.1
YP_407362.1
YP_407361.1
YP_407357.1
YP_407358.1
YP_687837.1
YP_687797.1
YP_687845.1
YP_687862.1
YP_688186.1
NP_708781.1
NP_838066.1
NP_838059.1
NP_838060.1
NP_838061.1
NP_838065.1
NP_838067.1
NP_838064.1
YP_403314.1
YP_403519.1
YP_403696.1
YP_404332.1
YP_404378.1
YP_405527.1
YP_405691.1
YP_405464.1
NP_308328.1
NP_309094.1
NP_309211.1
NP_309215.1
NP_309253.1
NP_310308.1
NP_311745.1
NP_312973.1
NP_312976.1
NP_312979.1
NP_312981.1
NP_312987.1
NP_312988.1
NP_312990.1
NP_312993.1
NP_312994.1
NP_312995.1
NP_312996.1
NP_312997.1
NP_312998.1
NP_312999.1
NP_313000.1
NP_313001.1
NP_313003.1
NP_313004.1

NP_313005.1
NP_313007.1
NP_286600.1
NP_286665.1
NP_286960.1
NP_286961.1
NP_286971.1
NP_287073.1
NP_287324.1
NP_287617.1
NP_290449.1
NP_290490.1
YP_668902.1
YP_669056.1
YP_669057.1
YP_669064.1
YP_669820.1
YP_670480.1
YP_670481.1
YP_670592.1
YP_670599.1
YP_670693.1
YP_670698.1
YP_670701.1
YP_670891.1
YP_671643.1
YP_671702.1
YP_672388.1
YP_543983.1
YP_541686.1
YP_541688.1
YP_541656.1
YP_542356.1
YP_542357.1
YP_543184.1
YP_539261.1
YP_539262.1
YP_539263.1
YP_539325.1
YP_539329.1
YP_539911.1
YP_539912.1
YP_539913.1
YP_539914.1
YP_539915.1
YP_539916.1
YP_539917.1
YP_539952.1
YP_539963.1
YP_540153.1
YP_540154.1
YP_540485.1
YP_540503.1

YP_540735.1
YP_540738.1
YP_540740.1
YP_540741.1
YP_540742.1
YP_540743.1
YP_540744.1
YP_540745.1
YP_540746.1
YP_541641.1
YP_541652.1
YP_541655.1
YP_541915.1
YP_541916.1
YP_541917.1
YP_541952.1
YP_542005.1
YP_542186.1
YP_542187.1
YP_542190.1
YP_542193.1
YP_542699.1
YP_542700.1
YP_542701.1
YP_542784.1
YP_542785.1
YP_542786.1
YP_543286.1
YP_543287.1
YP_543288.1
YP_543403.1
YP_543404.1
YP_543528.1
YP_543529.1
YP_543530.1
YP_543697.1
YP_544039.1
NP_752267.1
NP_752653.1
NP_752866.1
NP_752867.1
NP_752868.1
NP_752869.1
NP_752875.1
NP_752881.1
NP_753127.1
NP_753184.1
NP_753578.1
NP_753597.1
NP_753599.1
NP_753600.1
NP_753782.1
NP_753783.1

NP_753784.1
NP_754291.1
NP_754396.1
NP_754738.1
NP_754775.1
NP_755025.1
NP_755087.1
NP_755270.1
NP_755472.1
NP_755508.1
NP_755511.1
NP_755556.1
NP_755557.1
NP_755558.1
NP_755979.1
NP_756371.1
NP_756372.1
NP_756374.1
NP_756375.1
NP_756387.1
NP_756395.1
NP_756399.1
NP_757069.1
